# Schauta-Amreich Operation vs Piver II Procedure with Pelvic Lymphadenectomy for Cervical Cancer

**Published:** 2013-12

**Authors:** Giovanni Larciprete, Ioannis Malandrenis, Giuseppe Di Pierro, Carlotta Montagnoli, Federica Rossi, Chiara Centonze, Alessandro Bompiani, Valentina Panetta, Edoardo Valli, Mario Segatore, Herbert Valensise, Elio Cirese

**Affiliations:** 1 Department of Obstetrics and Gynecology, Fatebenefratelli Isola Tiberina Hospital, Rome, Italy;; 2 AFaR Associazione Fatebenefratelli per la Ricerca, Rome, Italy;; 3 L’altrastatistica srl Consultancy & Training, Biostatistics office, Rome, Italy;; 4 Department of Obstetrics and Gynecology, Azienda Ospedaliera Regionale San Carlo, Potenza, Italy;; 5 Department of Obstetrics and Gynecology, Tor Vergata University, Rome, Italy

**Keywords:** Cervical cancer, Lymphadenectomy, Piver II Operation, Schauta Amreich Operation

## Abstract

**INTRODUCTION:**

The aim of this study was to compare two groups of patients with early stage cervical cancer who underwent either abdominal or vaginal surgery, in terms of post-operative findings and survival.

**MATERIALS AND METHODOLOGY:**

55 patients with diagnosed cervical cancer were retrospectively selected for this study. They were preoperatively staged according to FIGO criteria. Forty four patients had disease between stages Ib and IIa with no evidence of extra-pelvic lymph node involvement and 10 patients had stage ≥ IIb.

**RESULTS:**

Of the 55 patients, 17 had been operated by Schauta-Amreich radical vaginal hysterectomy and 38 by Piver type II abdominal hysterectomy. No significant statistical differences have been found between two groups about age (median age was 49 for Schauta and 54 for Piver *p*=0.494) and parity of the patients (Median parity was 2 (range: 0-5) for Piver II group and 1 (range: 0-4) for Schauta group (*p*=0.607)) and about histotype and stage of the cervical cancer (34 patients with squamous cell carcinoma among Piver II Group vs 16 patients from Schauta Group; 4 women with adenocarcinoma from Piver II Group vs 1 subject from the Schauta Group; *p* value 1.000). Among the two groups there were significant statistical differences regarding the mean operative time (86 ± 28 minutes for Vaginal surgery and 115 ± 31 minutes for Abdominal surgery, *p*=0.038) and the average hospital stay (8.65 ± 4.42 days for abdominal surgery and 5.65 ± 2.3 days for vaginal surgery, *p*=0.020). Significant statistical difference was reported as regarding adjuvant RT, increased in the Piver II group with respect to the Schauta group (22 vs 4 pts; *p*=0.028). The survival rate at 5-years was without significant difference between the two groups (23 patients frof Piver II Group vs 11 patients from Schauta Group, *p*=0.510).

**DISCUSSION:**

This study confirms the benefits of the Schauta-Amreich vaginal radical hysterectomy in terms of hospital stay, mean operative time and early complications.

**CONCLUSION:**

We believe that this surgery is a plausible alternative to radical abdominal hysterectomy and could be considered to be a valid approach for the treatment of patients with cervical neoplasms, but still randomized trials are needed on this topic with respect to the ethical issues involved.

## INTRODUCTION

Improved overall survival rates combined with the best quality of life should be the primary targets in any invasive cancer treatment. At present, for tumors less than 4 cm, the standard treatment options for early stages of cervical cancer (IB to IIA of The International Federation of Gynecology and Obstetrics -FIGO- stages) are radical abdominal hysterectomy and pelvic lymphadenectomy, vaginal radical hysterectomy with pelvic lymphadenectomy or external pelvic irradiation plus brachytherapy, as adequate (1). With the presence of positive nodes, positive parametria or positive surgical margins, adjuvant concurrent chemoradiation (5 FU + Cisplatin or Cisplatin alone) improves survival compared with pelvic irradiation alone (2, 3). Since a long time, it has been thought that the survival rate of patients with stages IB and IIA depends exclusively on the size of their tumors and not on treatment modality (4).

For many gynecologists primary surgical treatment is considered to be the method of choice for tumors that are less than < 4 cm (5). The benefits of surgical treatment include preservation of the ovarian function and preservation of the vaginal function (lubrication, avoidance of stenosis) (5). Radical hysterectomy for the early stages of cervical carcinoma employs two approaches: the classical abdominal procedure, known as the Piver II/III operation, and the vaginal approach, called the Schauta-Amreich operation. Both operations achieve similar surgical results. Both approaches, according to the Rutledge classification (6), follow a type II of radical approach whereby, the medial half of cardinal and sacrouterine ligaments are removed. The classical Schauta technique was by Amreich (more radical resection of the dorsal part of lateral parametria) and Stoeckel (more conservative for lesions less than 2cm) (5, 7). As lymph spread of cervical carcinoma is the main route of transmission of the disease (5), systematic lymphadenectomy has become universally accepted as part of the management of patients with cervical cancer (8) and induced progressive modifications to the classical Schauta technique. In 1958, the Indian gynaecologist Suborg Mitraadded pelvic lymphadenectomy through bilateral extraperitoneal abdominal incision after radical vaginal hysterectomy (9). This technique has since been modified by our group using a single Pfannenstiel incision instead of 2 pelvic incisions (10).

Other techniques include a laparoscopic retroperitoneal interiliac lymphadenectomy approach (11) and laparoscopic transperitoneal pelvic lymphadenectomy (12). Laparoscopic assisted radical vaginal hysterectomy (LAVRH) avoids multiple scars from the operation (13). Although abdominal and vaginal approaches are considered comparable in terms of safety and efficacy in different studies (14, 15), insufficient data are available to explain differences in long term patient survival between these two procedures.

The aim of this retrospective study was to compare two groups of patients with early stage cervical cancer who underwent either abdominal or vaginal surgery in our Gynecological Department, in terms of post-operative findings and survival.

## MATERIALS AND METHODS

### Selection and Description of Participants

Fifty five patients admitted in Fatebenefratelli Isola Tiberina Hospital in Rome between January 1^st^ 2001 and December 31st 2005 with diagnosed cervical cancer were retrospectively selected for this study. Patients were preoperatively staged using FIGO criteria. Forty four patients had disease between stages Ib and IIa with no evidence of extra-pelvic lymph node involvement and 10 patients had stage ≥IIb and had also received primary chemoradiation treatment to reduce tumor size before surgery (external beam radiation and concurrent weekly platinum chemotherapy). All patients had pre-operative clinical staging included the following specialized investigations: full physical examination, chest X-rays, CT/MRI scan, routine pre-operative blood tests, cystoscopy and sigmoidoscopy if symptomatic. All patients were operated in our Gynecological Department.

### Technical Information

Of the patients included in this retrospective analysis, 38 had undergone a Piver type II radical hysterectomy and 17 patients had undergone a Schauta-Amreich radical vaginal hysterectomy (16). Pelvic nodes dissection was laparoscopic or extraperitoneal as adequate. Pelvic extraperitoneal lymphadenectomy was performed according to Silver *et al*. (17), with the modifications to the abdominal incision as previously described (10). The whole procedure of both radical vaginal hysterectomy and pelvic extraperitoneal and laparoscopic lymphadenectomy can be seen in **supplemental video 1**. Abdominal hysterectomy was carried out according to classical Piver type II procedure. The indications for post-operative radiotherapy and adjuvant chemotherapy were based on pathological findings at histological examination, including the presence of lymph node metastases, positive surgical margins, parametrial extension, lymphovascular permeation, and invasion of more than two-thirds of the cervical wall thickness (2, 3, 18). All patients were booked for scheduled follow-up in our Obst/Gyn outpatient ambulatory, with gynecological visits (pelvic examination and Pap smear) every 3 months for the first two years. All patients were then subsequently followed up at 6 monthly intervals for a total of 5 years. Imaging examinations were performed every 6 months as requested (Pelvic US, CT or MR in specific cases).

### Statistics

Shapiro wilk test was performed to verify Normality assumption. Kolmogorov-Smirnov test was used to compare continuous variables between two groups and Fisher’s exact test was employed for frequency or categorical data. Student t test was performed where requested. Significance was set at a probability value of <0.05. STATA 12.0 was used for all analyses.

## RESULTS

Of the 55 patients enrolled in this study 17 had been operated by Schauta vaginal hysterectomy with or without modified extraperitoneal or laparoscopic lymphadenectomy and 38 by Piver type II abdominal hysterectomy with or without conventional open intrabdominal lymphadenectomy (Table 1). Patients range from 28 to 79 years and no differences have been found between two groups (median age was 49 for Schauta and 54 for Piver *p*=0.494). Median parity was 2 (range: 0-5) for Piver II group and 1 (range: 0-4) for Schauta group (*p*=0.607). All cases were classified according to the FIGO staging and according to histological type as indicated in charts. There were no significant differences between the two groups per histotype or per stage (Table 1).

**Table 1:** Type of operation, FIGO staging, Histopatology of the studied population

	Piver II		Schauta		*p* value
	n	%	n	%	
	37		17		

**Type of intervention**					
Hysterectomy	0	0	4	24	0.028
hysterectomy with oophorectomy	5	13	1	6	
Hysterectomy with lymphadenectomy	12	32	5	29	
Hysterectomy with lymphadenectomy and oophorectomy	21	55	7	41	
**FIGO**					
In Situ					0.521
Microinvasive	4	11	1	6	
IA	4	10	6	36	
IB	15	39	8	47	
IIA	5	13	1	6	
IIB	6	16	1	6	
IIIA	1	3	0	0	
IIIB	2	5	0	0	
**Histopathology**					
Squamous cell carcinoma	34	89	16	94	1.000
Adenocarcinoma	4	11	1	6	

**Figure 1:**
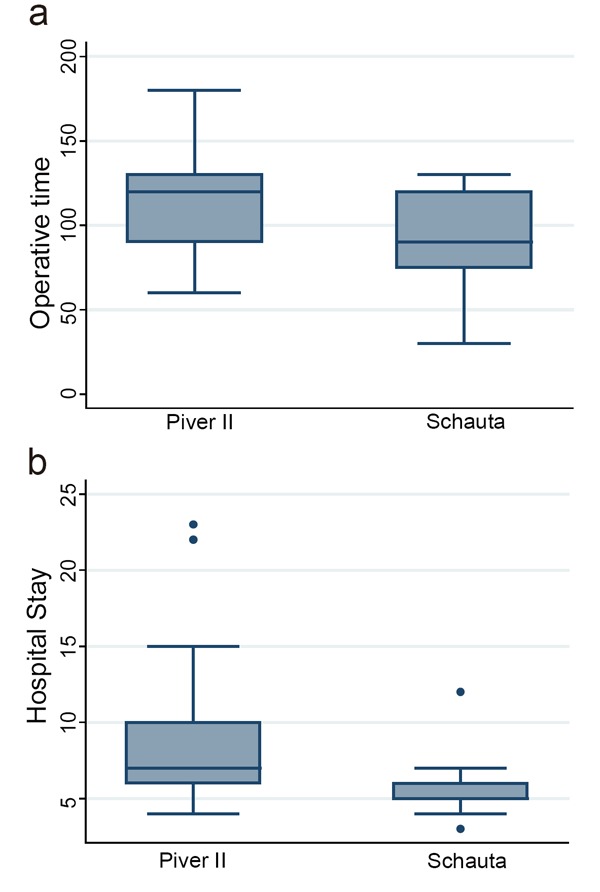
a, Operative time (minutes); b, Hospitalisation (days).

Regarding the intervention itself we showed that there was a significant difference in the mean operative time among the two groups (86 ± 28 minutes for Vaginal surgery and 115 ± 31 minutes for Abdominal surgery, *p*=0.038; Figure 1a); the average hospital stay for abdominal surgery was 8.65 ± 4.42 days and 5.65 ± 2.3 days for vaginal surgery with a significant difference (*p*=0.020) in the two groups (Figure 1b).

Preoperative values of Hb were respectively 12.56 ± 1.33 g/dl for the abdominal surgery and 13.12 ± 1.01 g/dl for vaginal surgery; postoperative values were respectively 9.84 ± 1.71 for abdominal and 10.99 ± 1.38 g/dl for vaginal procedures. Hb delta variation was not significantly different among groups (2.72 vs 2.13 g/dl; *p*=0.11). On the other side there was a difference for the necessity of transfusions in the two group, even if not statistically significative (10 transfusions in Piver II Group vs 1 transfusion in Schauta group; *p*=0.143).

The mean number of resected nodes was 18 (range 7-28) from the Piver II Group and 19 (range 8-25) from the Schauta Group, with no difference between the two groups of surgical treatment (*p*=0.256).

Regarding early complications, fever (temperature >38°C after two consecutive measurements 4 h apart from each other) was experienced in 8 patients undergoing Piver type II operation compared with no patient belonging to the Schauta group (*p*=0.048). No severe operative/post-operative complications occurred among the two groups (Table 2).

**Table 2:** Post-op. findings, recurrences, survival

	Piver II		Schauta		*p* value
	n	%	n	%	
	37		17		

Transfusion	10	26	1	6	0.143
Fever	0	0	8	47	0.048
Therapies associated with surgery					
*Adiuvant Chemotherapy*	17	45	4	24	0.210
*NACT*	7	18	0	0	0.084
*Adjuvant Radiotherapy*	22	58	4	24	0.028
*Neoadjuvant Radiotherapy*	3	8	0	0	0.543
Recurrences	8	21	3	18	1.000
*central Reccurrences*	5	13.2	3	17.6	0.692
*extrapelvic metastasis*	3	7.9	0	0	0.544
survival at 5 years	23	67.6	11	78.6	0.510
survival at 5 years in FIGO IIB	6	50	3	50	1.000

NACT, neo-adjuvant chemotherapy.

Significant difference was reported as regarding adjuvant RT, increased in the Piver II group with respect to the Schauta group (22 vs 4 pts; *p*=0.028; Table 2).

So far to date in this long term follow up interval, there were 8 central recurrences (5 from Piver II group and 3 from Schauta group, respectively) and 3 extrapelvic metastases only in Piver type II group.

At 5-years the 78.6% of patients undergoing Schauta vaginal hysterectomy and the 67.7% for patients undergoing Piver type II abdominal hysterectomy survived without significant difference (*p*=0.51). Stratificating survival rates on the basis of staging, and considering the IB stage, as more representative (23/54 patients), we obtained the same survival rates in the two groups (Table 2).

## DISCUSSION

Cervical cancer treatments must be effective and the treatment modality selected must ensure the best chance of improving mortality and morbidity rates. Historically, there was competition between surgeons who advocated radical vaginal hysterectomy without lymphadenectomy and those who supported radical abdominal hysterectomy with lymphadenectomy. The reduced postoperative complications and lower operative mortality rates mean that in some institutions the radical vaginal hysterectomy without lymphadenectomy (Schauta-Amreich operation) was undertaken more readily with some advocates.

In the 1965 Navratil stated that “*the indication for the Schauta operation must take the lymph node problem into accoun*t” and later he performed extraperitoneal pelvic lymphadenectomy, with the radical vaginal hysterectomy, as did Mitra (9).

More recently a new approach, with the introduction of the laparoscopically assisted vaginal radical hysterectomy (LAVRH), has become more popular. Querleu and Leblanc were credited with this approach (19).

Evaluation of lymph node status is an integral part of the surgical treatment for women with gynecologic cancer. It’s not possible to evaluate the pelvic lymph nodes by imaging alone and due to their deep anatomical location, most gynecologic cancers are staged at the time of surgery. Lymphadenectomy is important to achieve an adequate central dissection around the cervical tumor during the operation, so that involves removal of tissue from the hypogastric vessels, from the obturator fossa and from the lower presacral region. To date, several studies use to report a large number of newer approaches for lymphadenectomy.

The extraperitoneal pelvic lymphadenectomy can be considered an adequate technique to complement radical vaginal operation for cervical cancer since it represents the most cost-effective option due to shorter operating times, a greater number of nodes removed and reduced length of hospital stay. In a previous study, we compared the extraperitoneal lymphadenectomy versus laparoscopic technique in performing pelvic lymphadenectomy in a series of patients with locally advanced cervical cancer undergoing a radical vaginal hysterectomy (10). No difference was observed between the two groups, in terms of blood loss, postoperative pain, blood transfusion, hospital stay and postoperative complications. Thus, according to our previous data, extraperitoneal pelvic lymphadenectomy can be considered an adequate ancillary technique for radical vaginal operation for cervical cancer (10).

The present study compares two different approaches for the surgical treatment of cervical cancer, namely abdominal *vs* vaginal surgery. The number of lymph nodes resected was similar between the groups of patients, but there were significant differences in the mean operative time between the two groups, with reduced operating times in the vaginal operation and fewer patients with postoperative infection. Additionally, average hospital stay was significantly lower in the vaginal radical operation. According to the literature, our series of data show that radical vaginal hysterectomy carried out using the Schauta-Amreich technique with extraperitoneal lymphadenectomy represents adequate surgical management of early stage cervical cancer. This study confirms the benefits of the vaginal operation in terms of hospital stay, mean operative time and early complications. One of the major criticism against vaginal surgery in gynaecological oncology is about radicality and effectiveness.

We perfectly know that the present work is a poor retrospective observational study. Nonetheless we observed no differences between groups in terms of 5 years survival, even after stratification.

We believe that this surgery is a plausible alternative to radical abdominal hysterectomy and could be considered to be a valid approach for the treatment of patients with cervical neoplasms, but still randomized trials are needed on this topic with respect to the ethical issues involved.

Schauta operation is a poor technique indeed, with limited costs and limited impact on the woman’s body. Unfortunately the few surgeons able to do it in the past years, did not leave a flow school, did not let their coemployers to learn about this procedure. They had a rocket machine but they hide it as a personnel requirement, leaving spaces and open fields to the expensive laparoscopic and robotic procedures.
